# Initial gut microbiota composition is a determining factor in the promotion of colorectal cancer by oral iron supplementation: evidence from a murine model

**DOI:** 10.1186/s40168-025-02101-1

**Published:** 2025-04-21

**Authors:** Thibault Cuisiniere, Roy Hajjar, Manon Oliero, Annie Calvé, Gabriela Fragoso, Hervé Vennin Rendos, Claire Gerkins, Nassima Taleb, Marianne Gagnon-Konamna, François Dagbert, Rasmy Loungnarath, Herawaty Sebajang, Frank Schwenter, Ramses Wassef, Richard Ratelle, Éric De Broux, Carole Richard, Manuela M. Santos

**Affiliations:** 1 Nutrition and Microbiome Laboratory, Centre de Recherche du Centre hospitalier de l’, Université de Montréal (CRCHUM), Montréal, Québec Canada; 2 Institut du Cancer de Montréal, Montréal, Québec Canada; 3 Digestive Surgery Service, Centre Hospitalier de L’Université de Montréal (CHUM), Montréal, Québec Canada; 4 Department of Surgery, Université de Montréal, Montréal, Québec Canada; 5 Division of General Surgery, Université de Montréal, Montréal, Québec Canada; 6 Department of Medicine, Faculty of Medicine, Université de Montréal, Montréal, Québec Canada

**Keywords:** Colorectal cancer, Iron supplementation, Gut microbiota

## Abstract

**Background:**

Colorectal cancer (CRC) development is influenced by both iron and gut microbiota composition. While iron supplementation is routinely used to manage anemia in CRC patients, it may also impact gut microbiota and promote tumorigenesis. In this study, we investigated the impact of initial gut microbiota composition on iron-promoted tumorigenesis. We performed fecal microbiota transplantation (FMT) in *Apc*^*Min/*+^ mice using samples from healthy controls, CRC patients, and mice, followed by exposure to iron sufficient or iron excess diets.

**Results:**

We found that iron supplementation promoted CRC and resulted in distinct gut microbiota changes in *Apc*^*Min/*+^ mice receiving FMT from CRC patients (FMT-CRC), but not from healthy controls or mice. Oral treatment with identified bacterial strains, namely *Faecalibaculum rodentium*, *Holdemanella biformis*, *Bifidobacterium pseudolongum*, and *Alistipes inops,* protected FMT-CRC mice against iron-promoted tumorigenesis.

**Conclusions:**

Our findings suggest that microbiota-targeted interventions may mitigate tumorigenic effects of iron supplementation in anemic patients with CRC.

**Graphical Abstract:**

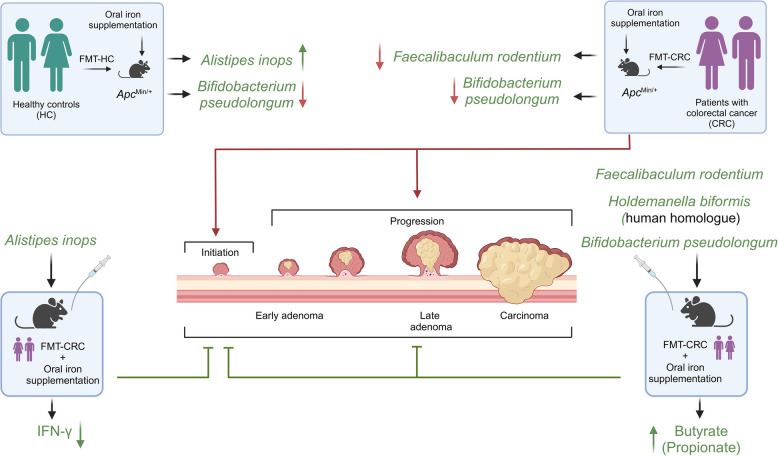

Video Abstract

**Supplementary Information:**

The online version contains supplementary material available at 10.1186/s40168-025-02101-1.

## Introduction

Colorectal cancer (CRC) is the third most common type of cancer, and the second leading cause of cancer-related deaths worldwide [1]. Geographical differences in the prevalence of CRC have been reported, most commonly associated with the so-called Western diet and lifestyle. A unique aspect of CRC is its close association with the gut microbiota, with research over the past decade establishing that imbalance in gut microbiota communities (dysbiosis) plays a pivotal role in the development and progression of CRC [2].

An emerging contributor associated with CRC is iron, both acquired as a dietary component or in the form of oral supplementation. Iron is essential to virtually all organisms, and maintaining iron homeostasis is critical as total iron deficiency is incompatible with life, whereas iron excess is dangerously toxic [3]. Besides being essential to the host, iron is also a limiting growth factor for most microorganisms that form the gut microbiota [4, 5]. In the present day, iron supplementation is broadly used to avoid iron deficiency. Despite its routine usage, there is increasing awareness of the deleterious effects caused by excess iron that have been mainly associated with the production of reactive oxygen species and their derived toxicity [3, 6]. In addition, iron is essential for DNA synthesis and contributes to cell cycle progression, making it fundamental for cellular proliferation and hence potentially fueling the growth of cancer cells [7].

Most CRC patients develop anemia which, in many cases, is the first symptom presented. Anemia in CRC may be due to iron deficiency likely compounded by anemia of chronic disease (ACD). ACD develops in patients with chronic conditions such as CRC [8] due to the immune system actively restricting iron absorption and availability within the host [9] through a process called nutritional immunity [10]. In addition, anemia in CRC may be exacerbated by bleeding in the gastrointestinal tract, further contributing to iron deficiency [11]. Management of anemia in CRC patients is pivotal as it is associated with poorer outcomes [12]. Treating anemia typically involves surgically removing the underlying cancer as well as addressing the iron deficiency itself [13], usually through oral iron supplementation in the form of ferrous sulfate [14]. However, most of the supplemented iron is not absorbed, passing into the colon where it becomes available for uptake by microorganisms [15], potentially affecting gut microbiota composition and function.

Accordingly, we and others have shown that dietary iron supplementation could negatively affect the composition and function of gut microbiota [16, 17]. In mice, iron supplementation was found to induce shifts toward a pro-carcinogenic gut microbiota during recovery from antibiotic exposure [18], while dietary supplementation with heme aggravated chemically induced colitis and promoted colorectal adenoma development [19]. Human studies have demonstrated a dose-dependent effect of iron supplementation on microbial communities. This effect is characterized by an increase in Proteobacteria and a decrease in beneficial taxa [20], while iron supplementation in children in developing countries promoted the growth of enteric pathogens, worsening gastrointestinal infections [21].

However, whether individuals with an imbalanced gut microbiota composition differentially respond to oral iron supplementation is presently not clear. This is particularly pertinent for patients with CRC, since mounting evidence indicates that intestinal dysbiosis allows for the colonization and proliferation of oncogenic bacteria. In this study, we investigated the effects of oral iron supplementation on gut microbiota and subsequent implications for intestinal carcinogenesis using *Apc*^*Min/*+^ mice transplanted with fecal microbiota from healthy controls and patients with CRC.

## Materials and methods

### Patient recruitment and sample collection

Colorectal cancer (CRC) patients scheduled for colorectal surgery were recruited through the Digestive Surgery Service at CHUM. CRC patients and healthy controls, matching for age and sex and with no prior antibiotic treatment in the last 6 months, were randomly enrolled (Supplementary Table S2). Fecal samples were collected from patients before their surgeries, adhering to the protocols established by the International Human Microbiome Standards project [22]. Samples were sealed in anaerobic conditions using hermetic containers and anaerobic sachets (BBL™ GasPak™ anaerobic indicator, Becton, Dickinson and Company, ON, Canada) and subsequently frozen at – 80 °C upon their arrival in the laboratory [23].

### Mice

Breeding colonies of WT and *Apc*^*Min/*+^ C57BL/6 mice (Jackson Laboratory, Bar Harbor, ME, United States) were established in a specific-pathogen-free (SPF) facility, and offspring were genotyped using allele-specific polymerase chain reaction (PCR) analysis, and the experiments were conducted as previously described [18, 24]. Mice were subjected to 2% DSS for 7 days, followed by a recovery period of 1 week. Prior to fecal microbiota transplantation, drinking water was supplemented with a mix of 3 antibiotics: ampicillin (1 mg/ml, WISENT Inc., QC, Canada), streptomycin (5 mg/ml, Sigma-Aldrich/MilliporeSigma Canada Co, ON, Canada) and colistin (1 mg/ml, SteriMax Inc., ON, Canada). Fecal microbiota transplantation was performed. Briefly, 200 μL of fecal material suspended in sterile 0.9% saline (Baxter, Mississauga, ON, Canada) at 100 mg/mL was administered by oral gavage and by applying 100 μL of the suspension to the animal’s fur, as previously described [23]. Mice received FMT from a single donor. Tumor assessment and immunocytochemistry were performed using well-established protocols as previously described [24, 25].

### Diets

Two weeks after FMT, mice were switched to either 50 ppm (Teklad TD.120515; Envigo, IN, USA) or 500 ppm (Teklad TD.120517; Envigo, IN, USA) iron diets for 4 weeks.

### Liver iron

Liver sample iron concentration was measured by ferrozine method using the QuantiChrom™ Iron Assay Kit (BioAssay Systems, CA, USA) according to the manufacturer’s protocol.

### Bacteria and culture conditions

*F. rodentium* (type strain no. 103405), *A. inops* (type strain no. 28863), *H. biformis* (type strain no. 3989) and *B. pseudolongum* (type strain no. 20094**)**, were purchased from the DSMZ (Deutsche Sammlung von Mikroorganismen und Zellkulturen) and grown on tryptone soy agar plates with 5% sheep blood (Oxoid, Thermo Fischer Scientific inc., ON, Canada) at 37° C in anaerobic conditions for 48 h. Bacterial colonies were scraped and suspended in sterile 0.9% saline (Baxter) for same-day administration by gavage. Control mice received sterile 0.9% saline only. Bacterial culture identification was monitored by real-time quantitative polymerase chain reaction (qPCR) using primers listed in Supplementary Table S3 and by matrix-assisted laser desorption ionization time-of-flight (MALDI-TOF) mass spectrometry. Bacterial presence in mouse fecal samples was confirmed by qPCR. Primers were designed with Primer-BLAST tool, PCR products were verified following sanger sequencing, and sequencing was confirmed with blast searchagainst public databases. Spent supernatants were obtained by centrifugation and filtration using a 0.2-μm filter of 48 h bacterial cultures grown under anaerobic condition in tryptone soy broth supplemented with 5% sheep blood.

### Cell lines

The human colonic adenocarcinoma cell line HT29 (ATCC® HTB- 38™; RRID: CVCL_0320) was a gift from Dr. Petronela Ancuta, (Department of Microbiology and Immunology, Université de Montréal) and was authenticated using short tandem repeat profiling and by examination of morphology and proliferation in vitro. The primary human tumor cell line was tested for mycoplasma contamination on a 7-day culture of the last passage using a universal mycoplasma PCR detection kit (abm®). Individual cryovials were thawed and cells used between the 15 th to 25 th passage. Cells were grown in McCoy’s 5 A (Modified) medium (Gibco, USA) supplemented with 10% Fetal Bovine Serum (FBS; Gibco, USA) and maintained in 75 cm^2^ culture flasks at 37 °C in a 5% CO_2_ (v/v) incubator in a humidified atmosphere. To evaluate the effect of short-chain fatty acid (SCFA) or the bacterial spent medium, 1.6 × 10^3^ cells were seeded overnight then stimulated with 50 mM sodium acetate (S5636, Sigma-Aldrich), 10 mM sodium propionate (P5436, Sigma Aldrich), 2 mM sodium butyrate (ARK2161, Sigma-Aldrich) or 20% v/v of the spent supernatant diluted in the cell culture medium for 48 h. MTT assays were performed and absorbance values were measured at a wavelength of 570 nm.

### Preparation and stimulation of splenocytes in-vitro

Spleens were removed aseptically, single cell suspensions were passed through a sterile 70-µm mesh (Thermo Fisher Scientific, CA, USA), and erythrocytes were lysed (Sigma, St. Louis, MO, USA). Cells were placed in complete antibiotic-free RPMI 1640 medium (Wisent, CA) supplemented with 10% FBS (Gibco, USA), 100 units/ml penicillin and 100 μg/ml streptomycin at a concentration of 4 × 10^6^ cells per ml. Cells were stimulated with 5% v/v of spent supernatant for 48 h with 1 µg/ml lipopolysaccharide (LPS) from *Escherichia coli* O55:B5 (Sigma-Aldrich/MilliporeSigma Canada Co.).

### Cytokine quantification, short-chain fatty acids, and real-time polymerase chain reaction

Inflammatory cytokines were quantified in colonic homogenates using a multiplex assay (Meso Scale Diagnostics, Rockville, MD, USA) or mouse IFN-γ ELISA MAX™ Deluxe kit (Biolegend, #430,804) and were corrected for protein concentration [23, 26, 27]. Short-chain fatty acid, and real-time polymerase chain reaction were performed as previously described [18, 26]. Briefly, short-chain fatty acids were measured in stool by LC–MS/MS using a method modified from Han J et al. [28]. Samples of approximately 20 mg were homogenized manually in 50% aqueous acetonitrile (30 µL per mg sample) using a polypropylene pestle and mixed thoroughly for 5 min at 4 °C. After centrifugation at 20,000 × *g*, 15 min at 4 °C, 30 µL of supernatants, blanks and standards were transferred to glass tubes with 10 µL of a 50% aqueous acetonitrile solution containing deuterated internal standards (acetic acid-d4, propionic acid-d5, butyric acid-d2, isobutyric acid-d7, valeric acid-d2, isovaleric acid-d2, hexanoic-d3 (CDN Isotopes, Pointe-Claire, QC, Canada)). Carboxyl groups were derivatized using 3-nitrophenylhydrazine. Derivatized organic acids were separated by reversed-phase chromatography (Nexera X2, Shimadzu) using a C18 column (Poroshell 120 EC-C18, 2.1 × 75 mm, 2.7 µm, Agilent) and detected by ESI–MS/MS in negative-ion mode (QTRAP 6500, SCIEX).

### 16S rRNA gene sequencing and analysis

DNA extraction and 16SrRNA sequencing and analysis was performed as previously described [26]. Primers for 16S rRNA gene amplification targeted the V5-V6 region: P609D 5′-GGMTTAGATACCCBDGTA- 3′ and P699R 5′-GGGTYKCGCTCGTTR- 3′. PCR amplification and sequencing were performed by Génome Québec and the MiSeq250 platform was used for 2 × 250 bp paired-end sequencing of PCR products. Amplification was performed using the following conditions: initial denaturation at 94 °C for 2 min, denaturation at 94 °C for 30 s, annealing at 58 °C for 30 s, extension at 72 °C for 30 s, final extension at 72 °C for 7 min, 4 °C hold over 35 cycles. Reagent controls were below the detection limit used by Génome Québec for quality assurance. 16S rRNA gene sequencing analysis was performed as previously described [18]. Briefly, forward and reverse demultiplexed 16S rRNA reads were processed using the Dada2 package (version 1.16) [29] in R (version 4.0.1), where they were denoised, filtered for chimeras, and clustered into sequence variants. Reads were trimmed at the first instance of a quality score of two or lower, or removed if they contained ambiguous nucleotides (N) or if two or more errors were expected based on the quality of the trimmed read. An average of 35,543 (± 780 SEM) high quality 16S rRNA sequences were obtained per sample. Amplicon sequence variants (ASVs) were assigned taxonomy using DADA2’s native naïve RDP Bayesian classifier against Silva training set v138.1 [30] and species taxonomy was assigned when 100% identity was found. Alpha and beta-diversity were assessed using the Phyloseq package (version 1.32.0) [31]. Statistical analysis of beta-diversity distances between groups were conducted with the vegan R package (version 2.5). Differential abundance analysis of the bacterial relative abundance was conducted using the DESeq2 R package (version 1.34.0). FDR correction was applied following the Benjamini–Hochberg procedure. LEfSe analysis was performed to identify differentially abundant species due to its ability to effectively manage sparse datasets [32], with the built-in function of the R package MicroEco [33]. Metagenomic inferences were performed using the tax4fun2 R package [34]. In experiments with FMT, in order to prevent overestimation of associations due to pseudo-replication, the biological unit consisted of the donor (*N* = 1 donor) and mice colonized from the same donor were considered replicates [35]. Therefore, to account for correlations between measurements of mice receiving FMT from the same donor, generalized estimating equations (GEE) were applied by the use of the geeglm function from the geepack R package [36] (version 1.3–2) with the family parameter set to “gaussian” or “binomial” and the correlation structure was specified as “independent”. Significance between parameters between groups was assessed with a Wald test. Metabolic pathway inference was performed using the NJC19 large-scale metabolic interaction network of the mouse and human gut microbiota [37]. To assess taxon-specific contributions to inferred metabolic pathways, we used metagMisc R package [38], which estimates the relative abundance-weighted contribution of each taxon to a given functional pathway predicted from the NJC19 network. Graphical representations were created with ggplot2 R package (version 3.4.2) and GraphPad Prism (version 7.00).

### Beta-diversity and constrained ordination analysis

To assess differences in microbial community structure across groups, we performed both Principal Coordinates Analysis (PCoA) and distance-based redundancy analysis (db-RDA) using Bray–Curtis dissimilarity metrics. The Bray–Curtis distances were calculated based on amplicon sequence variant (ASV) relative abundances. For db-RDA, we included the experimental groups (e.g., FMT-HC- 50 ppm, FMT-HC- 500 ppm, FMT-CRC- 50 ppm, FMT-CRC- 500 ppm) as explanatory variables to quantify the variance in microbial composition attributable to both the FMT source and iron supplementation levels. Statistical significance was assessed using permutational multivariate analysis of variance (PERMANOVA) with 999 permutations.

## Results

### Oral iron supplementation promotes carcinogenesis in Apc^Min/+^mice receiving fecal microbiota transplantation from patients with CRC

**Fig. 1:**
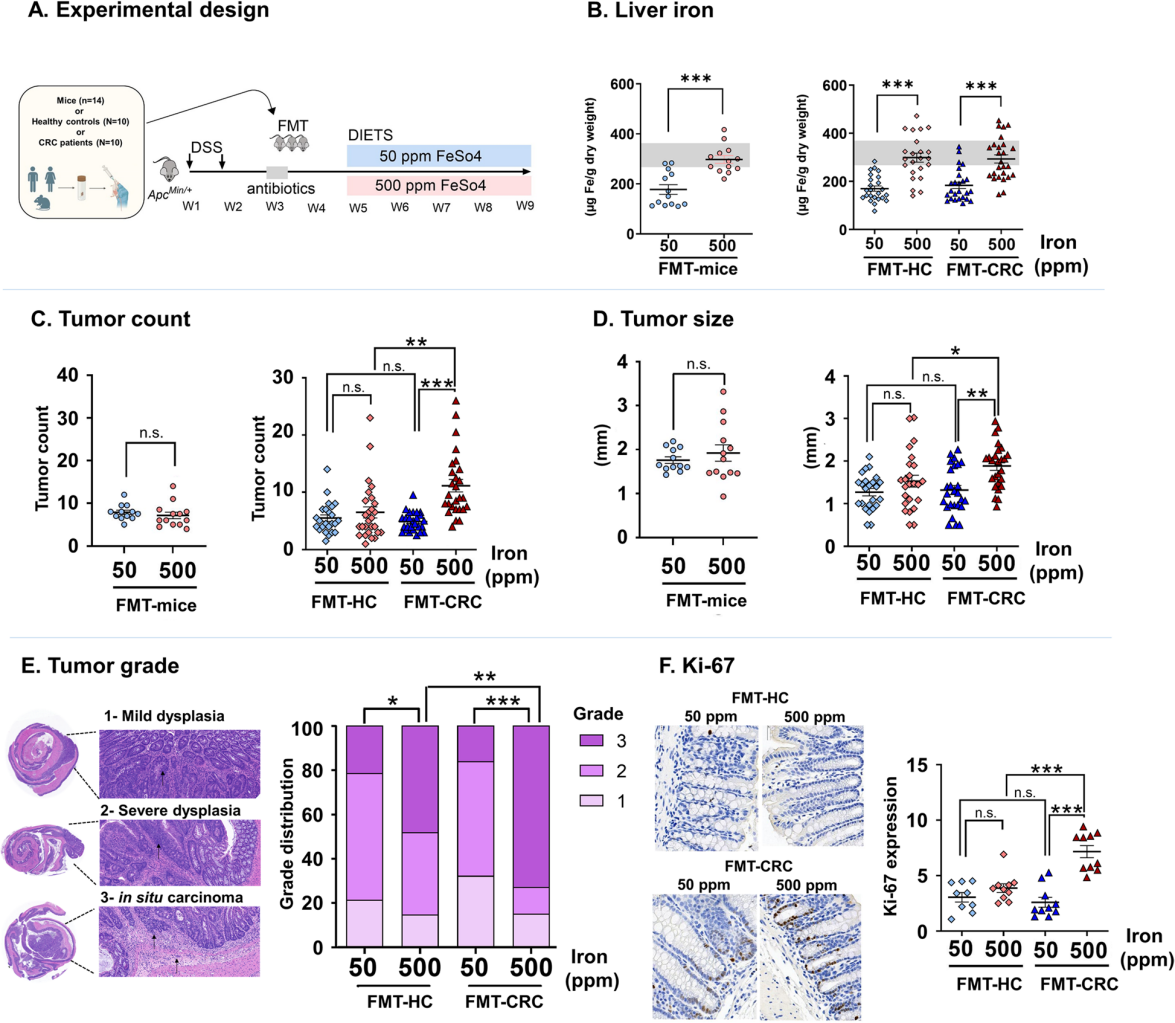
Dietary iron supplementation promotes colorectal carcinogenesis in *Apc*^*M*in/+^ mice transplanted with fecal microbiota (FMT) from patients with colorectal cancer (FMT-CRC) but not from healthy controls (FMT-HC) or murine donors. **A** Experimental design. **B** Iron concentration in the liver. Grey bars represent normal range for C57BL/6 WT mice. **C** Colorectal tumor counts and **D** tumor size. **E** Hematoxylin and eosin staining for determination of tumor grade. **F** Immunohistochemical staining for Ki- 67 quantification. Each symbol represents one mouse, FMT-mice (*n* = 13 (50 ppm); *n* = 13 (500 ppm)), FMT-HC (*n* = 24 (50 ppm); *n* = 26 (500 ppm)) and FMT-CRC (*n* = 25 (50 ppm); *n* = 27 (500 ppm)): 3 mice per donor, 10 donors per group. Bars are means ± SEM. *P* values were obtained using student* t*-test for FMT-mice. For mice transplanted with human fecal samples (FMT-HC and FMT-CRC) the generalized estimating equations (GEE) to correct for covariance structure of mice from a same donor (*n* = 3 mice/donor) was used. *P < 0.05, **P < 0.01, ***P < 0.001, n.s.: non-significant

To investigate the impact of the gut microbiota on colorectal carcinogenesis in response to oral iron supplementation, we conducted a study in antibiotic-conditioned *Apc*^*Min/*+^ mice receiving fecal microbiota transplantation (FMT) from either healthy controls (FMT-HC) or CRC patients (FMT-CRC). Mice transplanted with murine gut microbiota (FMT-mice) were used as controls. Two weeks after FMT, mice were placed on an iron sufficient diet (50 ppm) or iron excess diet (500 ppm) for 4 weeks (Fig. 1A). Demographic, clinical, and perioperative data of HC and CRC donors are shown in Supplementary Table S1 and Supplementary Table S2.

Mice receiving oral iron supplementation had significantly increased liver iron stores independently of FMT source, as shown in Fig. 1B. In addition, and as previously shown [39], iron supplementation induced significant increases in duodenal tumor count in all *Apc*^*Min/*+^ mice, independently of the FMT donor (Supplementary Fig. S1). Remarkably, iron supplementation promoted colorectal carcinogenesis in FMT-CRC, but not in FMT-HC mice, as shown by increased tumor count and burden (Fig. 1C, Supplementary Fig. S1, Supplementary Fig. S2; *P* < 0.001), tumor size (Fig. 1D; *P* < 0.01), higher incidence of in situ carcinomas (Fig. 1E; *P* < 0.001), and enhanced expression of the proliferation marker Ki- 67 in normal colon tissue (Fig. 1F; *P* < 0.001, Supplementary Fig. S1).

Since no differences were seen between FMT-HC and FMT-CRC mice kept on the iron sufficient diet (Fig. 1C–F), these results indicate that the pre-treatment gut microbiota composition determines the potential for iron supplementation to promote colorectal carcinogenesis. In contrast, iron supplementation in the duodenum promoted tumorigenesis independently of basal microbiota composition.

### Alterations in gut microbiota composition in response to iron supplementation differ between mice receiving fecal microbiota transplantation from HC or CRC donors

**Fig. 2:**
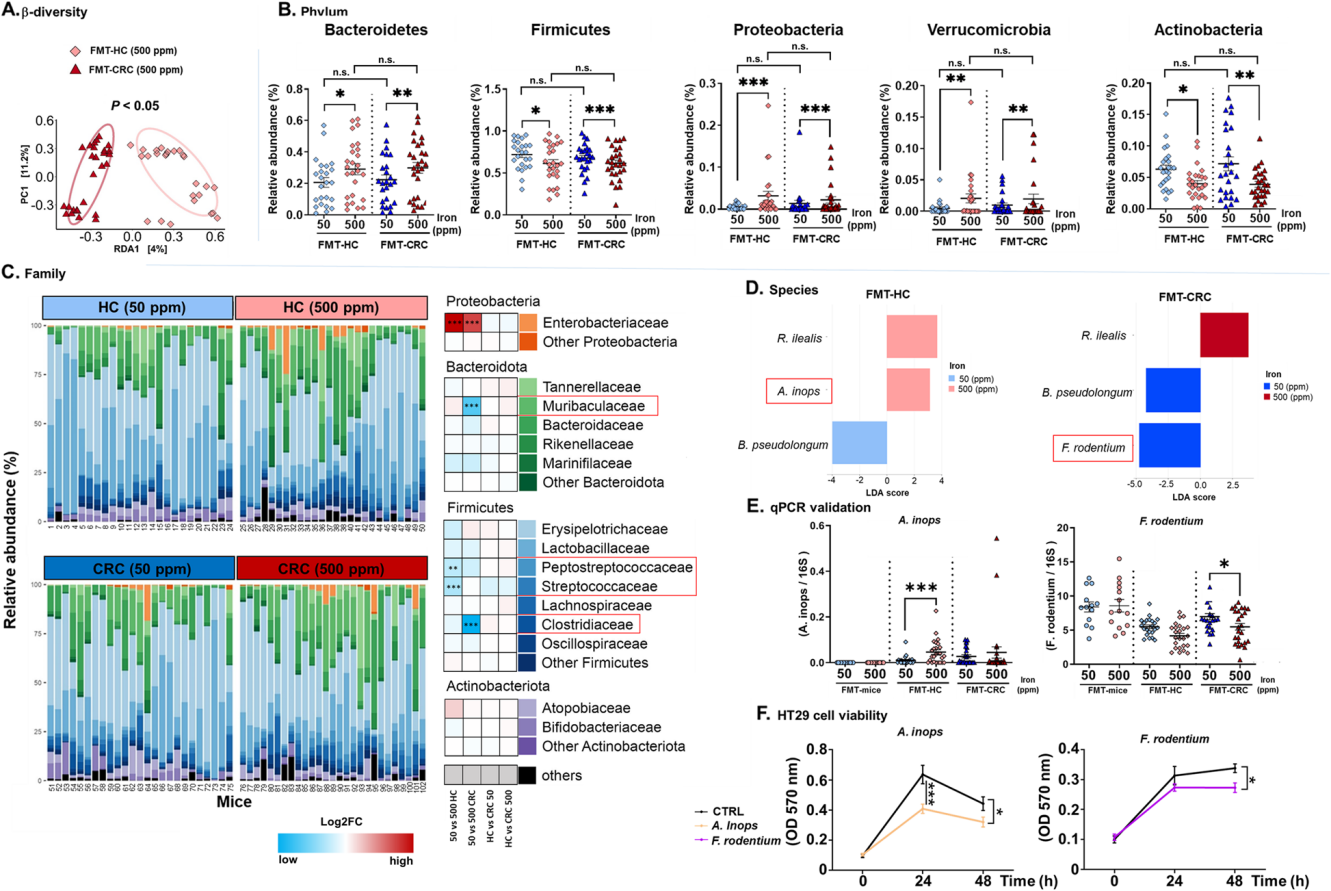
Iron supplementation differentially alters gut microbiota in** Apc**^*Min/*+^ mice receiving fecal microbiota transplantation from healthy controls (FMT-HC) and patients with colorectal cancer (FMT-CRC).** A** Distance-based redundancy analysis (RDA) of gut microbiota composition in fecal samples comparing the effect of dietary iron between FMT-HC and FMT-CRC mice. Relative abundances of microbial taxa at **B** phylum and **C** family levels. Significantly affected families after FDR correction exclusively in FMT-HC or FMT-CRC mice are framed in red. **D** Differential bacterial species between FMT-HC and FMT-CRC mice fed an iron sufficient (50 ppm) and iron excess (500 ppm) diet. (LEfSe, LDA > 3.0 and *P* < 0.05, FDR corrected). **E** *Alistipes inops*, and *Faecalibaculum rodentium* levels quantified by real-time PCR. **F** Proliferation of HT29 cells treated with culture broth (CTLR) or cell-free supernatants from *A. inops* or *F. rodentium* cultures. Bars are means ± SEM of *n* = 5 independent experiments. **B**, **E** Each symbol represents one mouse, FMT-mice (*n* = 13 (50 ppm); *n* = 13 (500 ppm)), FMT-HC (*n* = 24 (50 ppm); *n* = 26 (500 ppm)) and FMT-CRC (*n* = 25 (50 ppm); *n* = 27 (500 ppm)). Bars are means ± SEM. *P* values were obtained using student* t*-test for FMT-mice. For mice transplanted with human fecal samples (FMT-HC and FMT-CRC) the generalized estimating equations (GEE) to correct for covariance structure of mice from a same donor (*n* = 3 mice/donor) was used. *P < 0.05, **P < 0.01, ***P < 0.001, n.s.: non-significant

To investigate the association between gut microbiota alterations in response to iron supplementation and colorectal tumorigenesis, we performed 16S rRNA gene sequencing analysis of fecal samples from mice that received FMT from HC or CRC donors. We first confirmed the adequate transfer of the microbial ecosystem from donors to mice (Supplementary Fig. S3). Interestingly, CRC patients have distinct gut microbiota composition compared to HC, characterized by a significantly lower diversity (Supplementary Fig. S3). Analysis of the Bray–Curtis distances at the phylum, family, genus, and ASV levels revealed significant increases in microbial community dissimilarity as a consequence of iron supplementation, particularly in FMT-HC mice at each level analysed (*P* < 0.001), and at phylum and ASV levels in FMT-CRC mice (Phylum: *P* < 0.01; ASV: *P* < 0.05; Supplementary Fig. S4). In addition, constrained β-diversity analysis (distance-based redundancy analysis) revealed significant differences between bacterial communities of FMT-HC and FMT-CRC mice exposed to excess iron (*P* < 0.05; Fig. 2A, Supplementary Fig. S5), while α-diversity indices remained similar (Supplementary Fig. S6). Differential abundance analysis revealed significant changes in these microbial communities (Fig. 2B, C and Supplementary Table S4). Iron supplementation had consistent effects on phyla abundance (8 phyla were detected, Fig. 2B) independently of FMT donor (HC or CRC), characterized by a general increase in Bacteroidetes, Proteobacteria, and Verrucomicrobia. Concomitantly, Firmicutes and Actinobacteria were significantly decreased. Differences in response to iron supplementation between FMT-HC and FMT-CRC emerged at the family level (48 families detected, Fig. 2C) with significant decreases observed in *Peptostreptococcaceae* (*P* < 0.01) and *Streptococcaceae* (*P* < 0.001) in FMT-HC mice, while FMT-CRC mice had reduced *Muribaculaceae* (*P* < 0.001) and *Clostridiaceae* (*P* < 0.001) families. An expansion of *Enterobacteriaceae* was additionally observed in both FMT-HC and FMT-CRC mice receiving excess iron (*P* < 0.001).

LEfSe analysis at the species level (61 species identified, Fig. 2D) revealed that iron supplementation promoted the expansion of *Romboutsia ilealis* (Firmicutes phylum; *Peptostreptococcaceae* family) and the reduction of *Bifidobacterium pseudolongum* (Actinobacteria phylum; *Bifidobacteriaceae* family) in mice receiving FMT from either HC or CRC donors. Most importantly, iron supplementation resulted in the expansion of *Alistipes inops* (Bacteroidetes phylum; *Rikenellaceae* family) exclusively in FMT-HC mice, while *Faecalibaculum rodentium* (Firmicutes phylum; *Erysipelotrichaceae* family) significantly decreased in FMT-CRC mice only, suggesting potential protective effects. As seen in Fig. 2E and Supplementary Fig. S7, when quantified by real-time PCR, *A. inops* was indeed higher only in FMT-HC mice receiving the iron excess diet (*P* < 0.001) while *F. rodentium* levels were significantly lower exclusively in iron supplemented FMT-CRC mice (*P* < 0.05), further confirming 16S rRNA sequencing data. Notably, *A. inops* remained undetected in autologous FMT mice, indicating that its introduction and subsequent colonization of the mouse colon was of human origin. Next, we tested potential anticancer effects of *A. inops* and *F. rodentium* using the human colon adenocarcinoma cell line HT29. As shown in Fig. 2F, both *A. inops* and *F. rodentium* cell-free supernatant significantly inhibited the growth of HT29 cells after 48 h (*P* < 0.05).

### Iron supplementation differentially alters gut microbial metabolic pathways in mice receiving fecal microbiota transplantation from HC or CRC donors

**Fig. 3:**
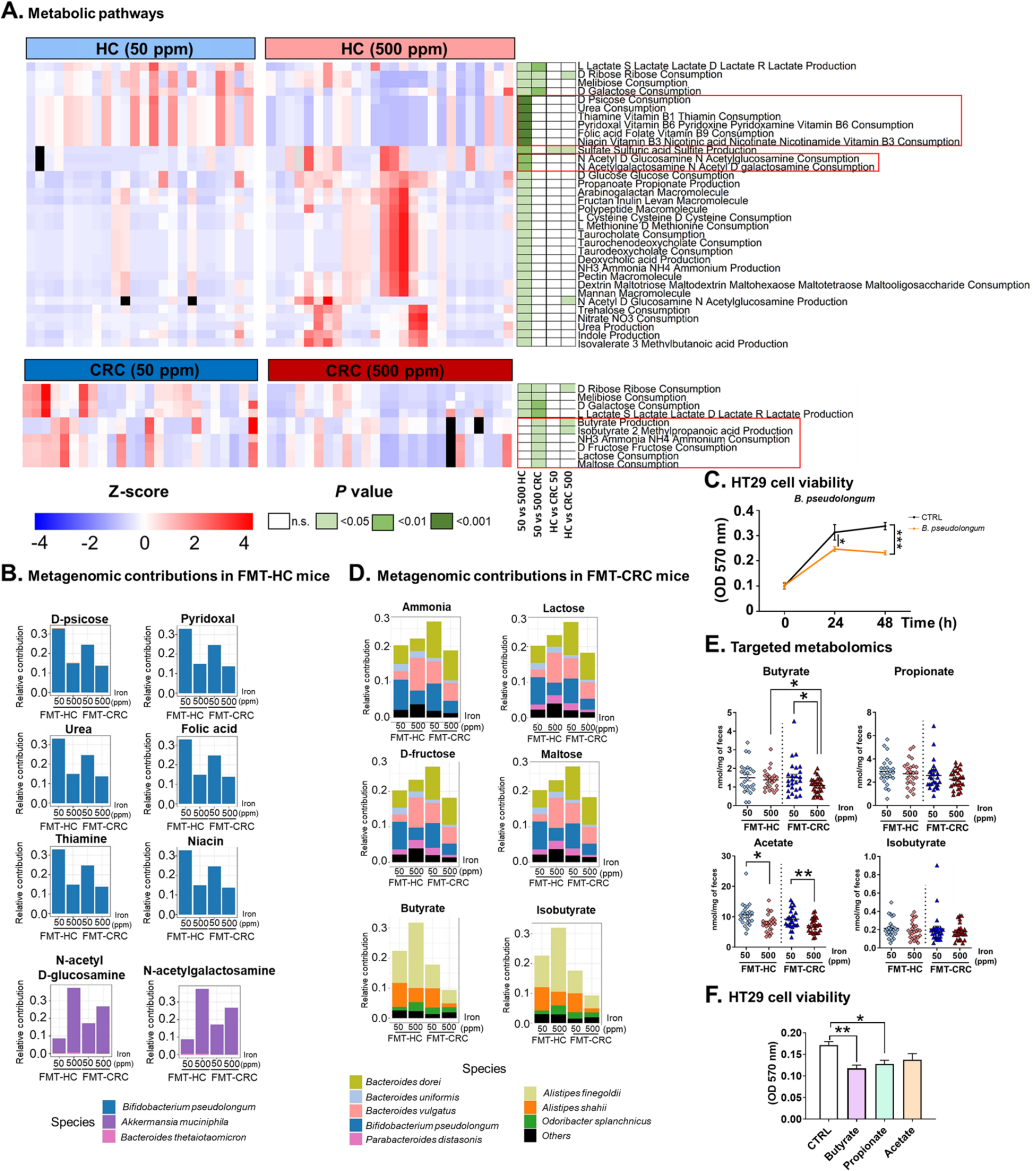
Iron supplementation differentially affectsmetabolic pathways and bacterial metagenomic contributionsin** Apc**^*Min/*+^ mice receiving fecal microbiota transplantation from healthy controls (FMT-HC) and patients with colorectal cancer (FMT-CRC).** A** Heatmap of differential metabolic pathways significantly affected among FMT-HC and FMT-CRC mice fed iron sufficient (50 ppm) or iron excess (500 ppm) diets. Outliers, defined as 8 times the mean of the group, are indicated in black. Significantly affected pathways families exclusively in FMT-HC (*P* < *0.01*) or FMT-CRC mice (*P* < 0.05) after FDR correction are framed in red. **B**, **D** Stacked bar charts showing the metagenomic contributors to the significantly affected metabolic pathways in **B** FMT-HC mice and **D** FMT-CRC mice fed the iron excess diet. **C** Proliferation of HT29 cells treated with culture broth (CTLR) or cell-free supernatant from *B. pseudolongum* cultures. *n* = 5 independent experiments. **E** Quantification of fecal butyrate, propionate, acetate, and isobutyrate in FMT-HC and FMT-CRC mice. Each symbol represents one mouse, FMT-HC (*n* = 24 (50 ppm); *n* = 26 (500 ppm)) and FMT-CRC (*n* = 25 (50 ppm); *n* = 27 (500 ppm)). *P* values were obtained using the generalized estimating equations (GEE) to correct for covariance structure of mice from a same donor (*n* = 3 mice/donor). **F** Assessment of HT29 cell proliferation in the presence of the SCFAs (butyrate, propionate and acetate, *n* = 5). *P* values were obtained using one-way ANOVA and post-hoc Dunnett test. Bars are means ± SEM. *P < 0.05, **P < 0.01, ***P < 0.001, n.s.: non-significant

To further identify gut bacterial metabolic pathways and microbial contributions to metabolic pathways. modified by iron supplementation in our model, we performed a functional prediction analysis based on 16S rRNA profiles using NJC19, a large-scale literature-curated metabolic interaction network for the mammalian gut microbiota [37], and the *metagMisc* analysis tool [38], leading to the inference of 6 476 KOs and 306 pathways. We found striking differences in inferred metabolic functional potential in response to iron supplementation between FMT-HC (34 functions affected, of which 30 were unique) and FMT-CRC mice (10 functions affected, 6 unique), revealing considerably more pathways being affected in FMT-HC mice (Fig. 3A). In iron supplemented FMT-HC mice the most significant differential alterations occurred in pathways related to the consumption of D-psicose monosaccharides, urea, and vitamins pyridoxal, folic acid, thiamine, and niacin, which were significantly downregulated (Fig. 3A, *P* < 0.001). The contributions to these changes were predominantly attributed mostly to *Bifidobacterium pseudolongum* (Fig. 3B). Of notice, HT29 cells exposed to *B. pseudolongum* cell-free supernatant showed significantly reduced proliferation, indicating potential anti-carcinogenic effects (Fig. 3C, *P* < 0.001).

On the other hand, potential consumption of glucose and galactose-derived amines (N-acetyl D-glucosamine and N-acetylgalactosamine) was enriched (*P* < 0.01), and these metabolic activities were primarily associated with *Akkermansia muciniphila* (Fig. 3B). Conversely, in FMT-CRC mice, *Bacteroides vulgatus* and *Parabacteroides distasonis* (Fig. 3D) contributed most to the downregulation of the potential consumption of ammonia and the sugars lactose, D-fructose and maltose (Fig. 3A, *P* < 0.05). In addition, butyrate and isobutyrate production potentials in FMT-CRC were significantly reduced (Fig. 3D, *P* < 0.05), attributed to contributions from mostly *Alistipes finegoldii* and *Alistipes shahii*. Further targeted metabolomics analysis confirmed that fecal levels of butyrate, but not isobutyrate, in iron supplemented FMT-CRC mice were significantly lower compared to FMT-HC mice on the same diet (Fig. 3E; *P* < 0.05). In-vitro experiments using HT29 further demonstrate the anticarcinogenic effect of butyrate (*P* < 0.01) and propionate (*P* < 0.05), but not acetate (Fig. 3F).

These results indicate that, depending on initial gut microbiota composition, iron supplementation can differentially alter both gut microbiota composition and its associated functions. In addition, we identified two bacterial strains, namely *A. inops* and *F. rodentium*, that were differentially affected in iron supplemented FMT-HC *vs* FMT-CRC mice, and *B. pseudolongum,* that was reduced in all mice receiving iron supplementation.

### Faecalibaculum rodentium and its human homologue Holdemanella Biformis protect against iron-promoted carcinogenesis

**Fig. 4:**
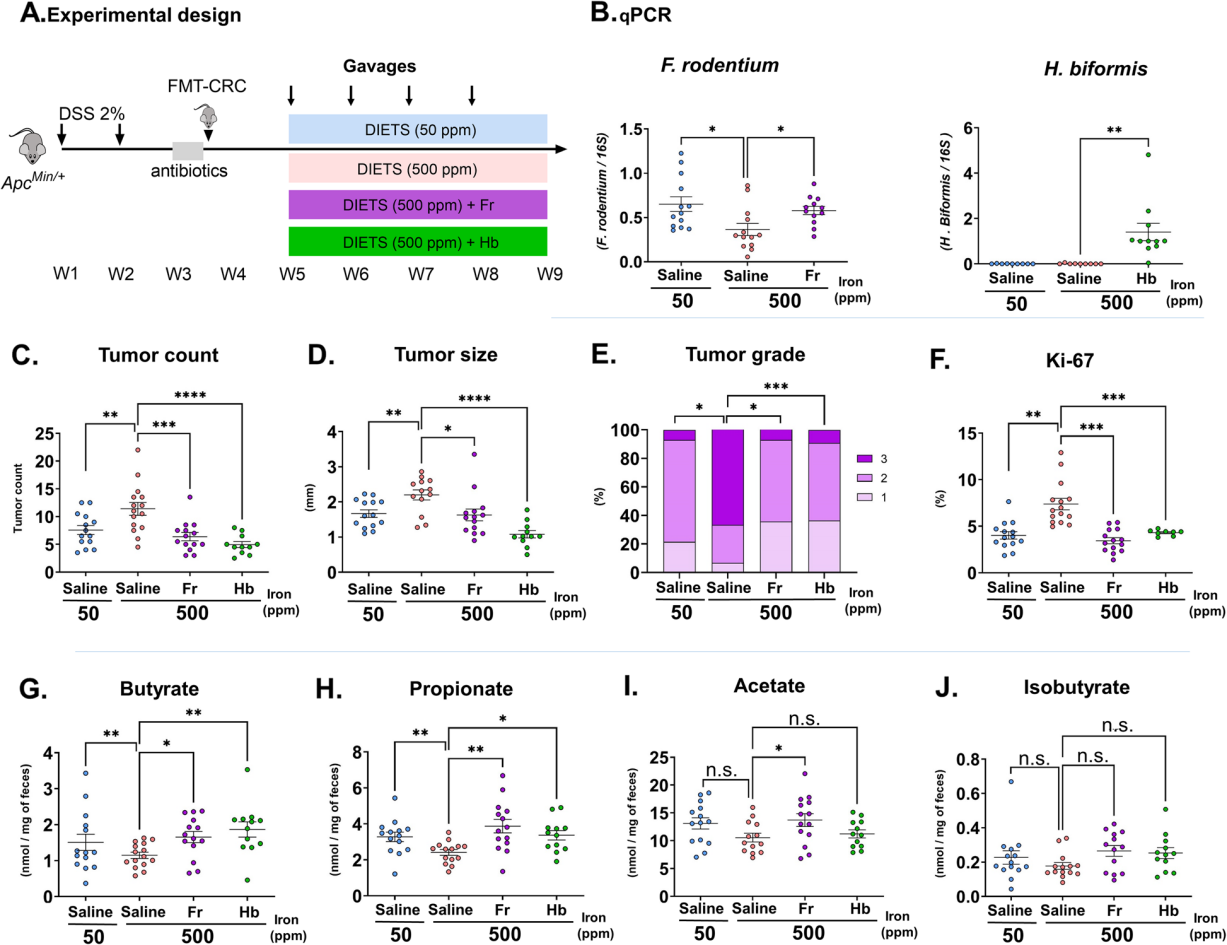
*F. rodentium* and *H. biformis* attenuate iron-promoted colorectal carcinogenesis in FMT-CRC *Apc*^*Min/*+^ mice.** A** Experimental design. **B** Fecal levels of *F. rodentium* (Fr) and *H. biformis* (Hb) quantified by real-time PCR in mice at endpoint. **C** Colorectal tumor count, **D** tumor size, **E** tumor grade, **F** colonic Ki- 67 quantification and fecal **G** butyrate, **H** propionate **I** acetate, and **J** isobutyrate concentrations in FMT-CRC mice. Each symbol represents one mouse with bars showing means ± SEM (*n* = 11–15); *P < 0.05, **P < 0.01, ***P < 0.001, n.s.: non-significant. *P* values were obtained using one-way ANOVA and post-hoc Dunnett test

To further explore the role of *F. rodentium*, which we found to be significantly reduced in iron supplemented FMT-CRC mice, but not in FMT-HC mice, in iron-promoted colorectal carcinogenesis, antibiotic-conditioned *Apc*^*Min/*+^ mice were colonized with gut microbiota from patients with CRC and placed on iron sufficient or iron excess diets. During exposure to the experimental diets, mice received either *F. rodentium* strain ALO17 (Fr) or saline by weekly gavage. Additionally, a 3rd mouse group received the *H. biformis* strain VPI C17 - 5 (Hb), the human homologue phylogenetically related to *F. rodentium *[40] (Fig. 4A). Quantitative PCR analysis of fecal samples collected at the end of the experiment showed a significant increase in the relative abundance of *F. rodentium* (*P* < 0.05) and *H. biformis* (*P* < 0.01) in mice gavaged with the bacterial strains (Fig. 4B). In line with the results shown in Fig. 1, enhanced colorectal carcinogenesis was seen in mice gavaged with saline that received the iron-supplemented compared with the iron sufficient diet (Fig. 4C–F). In contrast, iron-supplemented mice treated with *F. rodentium* or *H. biformis* presented with reduced colorectal tumor burden as evidenced by tumor count (Fig. 4C; *P* < 0.001), size (Fig. 4D; Fr: *P* < 0.05, Hb: *P* < 0.001), grade (Fig. 4E; Fr: *P* < 0.05, Hb: *P* < 0.001), and Ki- 67 (Fig. 4F; *P* < 0.001). The effects of *F. rodentium* and *H. biformis* were associated with increased fecal SCFAs, primarily butyrate and propionate, but not isobutyrate (Fig. 4G-J). These results suggest that *F. rodentium* ALO17 and *H. biformis* VPI C17 - 5 can mitigate iron-promoted tumorigenesis and increase SCFA production.

### Alistipes inops and Bifidobacterium pseudolongum reduce iron-induced colorectal tumorigenesis in mice transplanted with fecal microbiota from patients with colorectal cancer

**Fig. 5:**
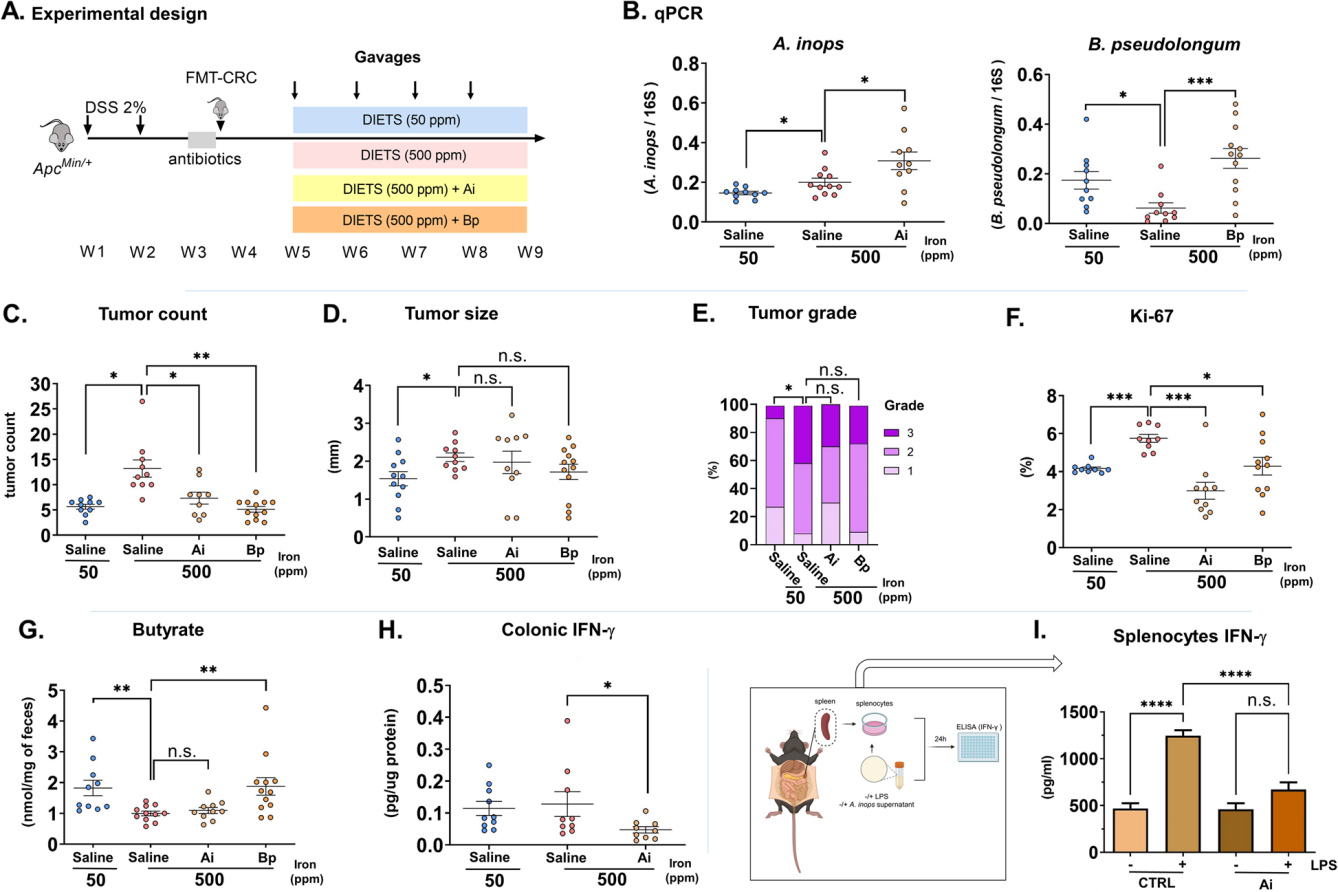
*A. inops* and *B. pseudolongum* reduce iron-induced colorectal tumorigenicity in FMT-CRC** Apc**^*Min/*+^ mice. **A** Experimental design. **B** Fecal levels of *A. inops* (Ai) and *B. pseudolongum* (Bp) quantified by real-time PCR in mice at endpoint. **C** Colorectal tumor count, **D** tumor size, **E** tumor grade, **F** colonic Ki- 67 quantification, and **G** fecal butyrate concentrations in FMT-CRC mice*.***H** levels of IFN-γ in the colonic tissue of mice. **I** IFN-γ ELISA quantification of splenocytes supernatants harvested at 24 h (*n* = 3). Each symbol represents one mouse. Bars are means ± SEM (*n* = 10–12); *P < 0.05, **P < 0.01, ***P < 0.001, ****P < 0.0001, n.s.: non-significant. *P* values were obtained using one-way ANOVA and post-hoc Dunnett test

Next, we investigated the potential *of Alistipes inops* strain 627, which significantly expanded in iron supplemented FMT-HC, but not in FMT-CRC mice, to revert iron-promoted tumorigenesis in FMT-CRC mice. We additionally evaluated the effect of *Bifidobacterium pseudolongum* strain 28 T given its prominent implication in metabolic changes (Fig. 3B) and their reduction by iron supplementation in both FMT-HC and FMT-CRC mice (Fig. 2D), further validated by qPCR (Supplementary Fig. S7). As previously, antibiotic-conditioned *Apc*^*Min/*+^ mice were colonized with gut microbiota from patients with CRC and placed on iron sufficient or iron excess diets while receiving weekly gavages of saline, *A. inops* or *B. pseudolongum* (Fig. 5A). At the end of the experiment, iron-supplemented FMT-CRC mice that received the bacterial supplementation had significantly increased fecal *A. inops* or *B. pseudolongum* loads compared to saline-treated mice (Fig. 5B, A*. inops*: *P* < 0.05; *B. pseudolongum*: *P* < 0.001). As expected (Fig. 1C) iron supplementation significantly increased *A. inops* and decreased *B. pseudolongum* in FMT-CRC mice (Fig. 5B, A*. inops*: *P* < 0.05; *B. pseudolongum*: *P* < 0.05). *A. inops* and *B. pseudolongum* supplementation were able to significantly reduce tumor count (*A. inops*: *P* < 0.05; *B. pseudolongum*: *P* < 0.01) and Ki- 67 (*A. inops*: *P* < 0.001; *B. pseudolongum*: *P* < 0.05), but not tumor size and grade in mice fed the iron excess diet (Fig. 5C–F). Furthermore, fecal butyrate, isobutyrate, acetate, and propionate remained unaffected by *A. inops* supplementation, while *B. pseudolongum* supplementation induced an increase in fecal butyrate (*P* < 0.01, Fig. 5G, Supplementary Fig. S8). Given the importance of inflammation in colorectal carcinogenesis [41], we questioned whether FMT-CRC mice supplemented with *A. inops* could have reduced colonic inflammation. In our analysis of colonic samples using a multiplex panel of five cytokines, we observed a significant reduction in IFN-γ concentration (*P* < 0.05, Fig. 5H, Supplementary Fig. S9). To further test the effect of *A. inops* on IFN-γ response*,* we exposed lipopolysaccharide-stimulated (LPS) murine splenocytes to supernatants from *A. inops* cultures (Fig. 5I). We found that exposure to *A. inops* culture supernatants significantly supressed LPS-induced IFN-γ production (*P* < 0.0001). These results suggest that both *A. inops* 627 and *B. pseudolongum* affect INF-γ and SCFAs production and mitigate iron-promoted carcinogenesis.

## Discussion

In this study, we provide evidence that the reported colorectal tumorigenic effects of oral iron supplementation depend on the initial gut microbiota composition. Using the spontaneous transgenic *Apc*^*Min/*+^ CRC model, we show that only mice transplanted with CRC-associated fecal microbiota have enhanced colorectal carcinogenesis promoted by iron supplementation. In contrast, iron supplementation did not affect colorectal tumor development in mice transplanted with fecal microbiota from healthy human donors or from mice kept on a standard diet.

Emerging evidence indicates that oral iron supplementation may significantly impact the progression of CRC through both direct and indirect mechanisms. Direct effects include modulation of iron-related proteins involved in local iron homeostasis leading to iron accumulation in colon tumors, which require high amounts of iron to sustain their proliferation [42]. In turn, iron accumulation in malignant cells leads to a robust increase in reactive oxygen species (ROS), which further contribute to colon tumorigenesis [43]. Additional pathways of iron induced-tumorigenesis involve oncogene activation, resulting in, for example, the aberrant activation of Wnt/β-catenin signaling [44], a hallmark of CRC [45]. Accordingly, luminal iron levels have been shown to significantly increase duodenal tumorigenesis in *Apc*^*Min/*+^ mice, highlighting the importance of iron for the survival and proliferation of Apc-deficient gut epithelial cells [46]. This previous study is in line with our findings that, in the duodenum, oral iron supplementation has pro-carcinogenic effects, regardless of the initial gut microbiota composition. Hence, duodenum susceptibility could be linked to its role as the primary site of dietary iron absorption [47].

Indirect mechanisms involved in iron-mediated CRC progression are linked to changes in gut microbiota composition and function. Of notice, the gut microbiota composition plays a critical role in the development of CRC [48]. In our study, mice receiving a sufficient iron diet (50 ppm) did not exhibit significant differences in colorectal tumorigenesis between FMT-HC and FMT-CRC groups, contrasting with previous reports showing CRC-FMT alone can promote tumor development [49]. This finding may be explained by the fact that standard rodent diets contain approximately 200 ppm iron [50]. An iron sufficient diet may be insufficient to amplify the pro-tumorigenic effects of CRC-associated gut microbiota. Here we show that the strong link between CRC dysbiosis and carcinogenesis in the colon is in fact dependent on iron availability in the luminal space. This finding highlights the need to re-evaluate anemia treatment options in the context of CRC.

We and others have previously shown that iron supplementation induces shifts in gut microbiota that, while smaller than those following antibiotic treatments, are consistent and persistent [17, 18]. More recent studies have found that iron-induced microbiota changes are individual-specific in healthy humans, with some participants responding substantially to iron supplementation while in others iron did not induce significant changes [51]. Our constrained beta-diversity analysis is in line with these studies as it demonstrates clustering in gut microbiota composition between mice receiving FMT from HC versus CRC patients caused by iron supplementation.

We further identified *F. rodentium* and *A. inops* as two species differentially altered in iron supplemented FMT-HC vs. FMT-CRC mice. *F. rodentium* was decreased in FMT-CRC mice, suggesting a potential beneficial effect. Indeed, we further showed that bacterial supplementation with *F. rodentium* or with its human homologue *H. biformis* was able to revert iron-induced CRC promotion. The beneficial effects of *F. rodentium* and *H. biformis* were associated with increased fecal butyrate levels, which were significantly decreased in iron-supplemented FMT-CRC mice. Our results are in line with previous studies reporting the ability of both species to produce butyrate [40]. The anticarcinogenic action of butyrate has been linked to its inhibition of histone deacetylase (HDAC) [52], promotion of apoptosis, and anti-inflammatory properties [53], which collectively contribute to the reduction of CRC cell proliferation [54–56].

In turn, FMT-HC mice showed a unique increase in *A. inops*, a newly proposed species [57] belonging to the *Alistipes* genus which has been associated with protective effects against colorectal tumorigenesis [58]. Unlike *F. rodentium*, *H. biformis*, and *B. pseudolongum*, *A. inops* did not significantly alter luminal colonic SCFAs levels under our experimental conditions. Nonetheless, *A. inops* was effective in attenuating iron-induced colorectal carcinogenesis and was associated with decreased colonic IFN-γ levels and reduced IFN-γ production by LPS-stimulated splenocytes in vitro. IFN-γ is a cytokine involved in inflammatory and immune responses [59, 60] and plays essential roles in cancer diseases, with both pro- and anti-carcinogenic activities being reported [61, 62]. Elevated IFN-γ has been implicated in CRC progression through the promotion of gp70 expression [63] resulting in the inhibition of infiltrating CD8^+^ T cells [64] and impairing the immune system’s ability to effectively target CRC cells [63, 65]. In addition, IFN-γ has been shown to indirectly promote angiogenesis [66], further promoting tumor growth.

Overall, the results show that the four identified bacteria have anti-carcinogenic properties related to dietary iron-mediated tumorigenesis, and do not exclude the possibility that their mechanisms of action may overlap, since SCFAs that increased in response to *F. rodentium*, *H. biformis* and *B. pseudolongum*, are also known to impact cytokine production, including IFN-γ [67]. Additional research is required to unveil further mechanistic insights into how these bacteria exert their protective effects, including comprehensive phenotypic analyses, and immune and epithelial cell profiling. Nevertheless, our present findings confirm the potential for microbiota-based interventions as a strategy for CRC prevention [68] and the mitigation of adverse consequences associated with oral iron supplementation.

In addition to changes in gut bacterial composition, we also identified gut bacteria metabolic pathways and metagenomic contributions modified by iron supplementation. This analysis revealed that the gut bacteria metabolic pathways of FMT-HC mice fed the iron supplemented diet were highly altered compared to FMT-CRC mice, possibly suggesting a stronger response to excess luminal iron, limiting its toxicity [69] and minimizing the shift toward a pro-carcinogenic gut microbiota. As such, iron-induced alterations in FMT-HC mice led to a reduction in the capacity for microbiota to consume vitamins pyridoxal, folic acid, thiamine, and niacin. These compounds are known to have protective roles in various cancers including CRC [70], and the downregulation of these metabolic pathways in bacteria may increase their availability for the host. The contributions to these metabolic changes were predominantly attributed to *B. pseudolongum*, which we further show to have anticarcinogenic potential both in vitro and in vivo. These results align with our gut microbiota compositional analysis, as *B. pseudolongum* was significantly decreased in mice fed the iron excess diet. Consistent with this, a recent study revealed that *B. pseudolongum* and its derived inosine have anti-tumorigenic effects [71] linked to the enhancement of antitumor T cells function [72]. Most importantly, these potentially adaptive changes in metabolic pathways were absent in iron supplemented FMT-CRC mice, in which a downregulation of the potential consumption of pro-carcinogenic compounds such as ammonia and the sugars lactose, D-fructose and maltose was instead observed, which may further enhance microbial tumorigenicity [73]. For example, a decrease in ammonia consumption would lead to more luminal ammonia. Ammonia is a potential carcinogenic product as the continuous exposure of colonocytes to free ammonia has been shown to contribute to CRC development [74] and to the promotion of T cell exhaustion [75]. While our study provides valuable insights into the relationship between iron supplementation, gut microbiota functions, and colorectal cancer promotion, it is important to acknowledge limitations to the metabolic pathway predictions [76]. Future work should be directed at exploring the complex bidirectional relationships between gut microbiota, iron metabolism, mucosal immunity, and CRC development and progression, and developing predictive frameworks based on gut microbial profiles. In fact, the differential response to iron supplementation observed between mice colonized with microbiota from healthy controls versus CRC patients highlights the potential for specific microbial taxa and/or functional signatures to serve as biomarkers of susceptibility to iron-promoted tumorigenesis.

**Fig. 6:**
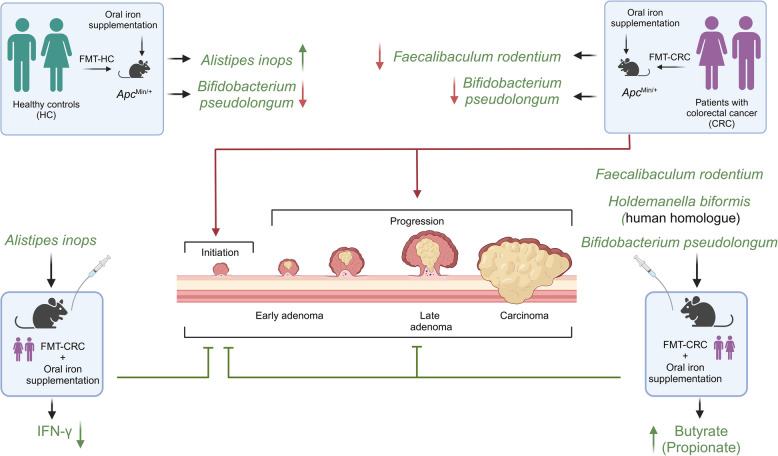
Graphical abstract summarizing the major findings of this study. Iron supplementation induces deleterious shifts in CRC-associated gut microbiota composition and function. Bacterial supplementation with *Alistipes inops*, *Bifidobacterium pseudolongum*, *Faecalibaculum rodentium* or *Holdemanella biformis* can revert iron-induced colorectal carcinogenesis. Green arrows and lines represent beneficial effects while red arrows and lines illustrate deleterious effects. Blunt lines represent inhibition. Created with BioRender.com

In conclusion, our study underscores the role of iron availability in gut bacterial community structure and function and contributes to a better understanding of the relationship between oral iron supplementation, the gut microbiota, and CRC promotion. Iron supplementation in the presence of CRC-associated gut microbiota could induce deleterious effects, thereby contributing to colorectal tumorigenesis. Most importantly, our findings show that microbiota-based approaches are promising to optimize oral iron therapy while avoiding deleterious and potentially pro-carcinogenic effects (Fig. 6). Further research is required to validate these findings in human subjects and to explore the translational potential of microbiota-based interventions for personalized anemia management and colorectal cancer prevention.

## Supplementary Information

Additional file 1. Supplementary Figure S1. Dietary iron supplementation promotes duodenal tumorigenesis in *Apc*^M^^in/+^mice independently of fecal microbiota transplant origin. Supplementary Figure S2: Representative pictures of the colon of *Apc*^M^^in/+^mice.Supplementary Figure S3: CRC associated gut microbiota differ from healthy control gut microbiota. Supplementary Figure S4: Bray-Curtis distances. Supplementary Figure S5: Beta diversity analysis of FMT-HC and FMT-CRC as a function of the dietary iron and initial gut microbiota composition. Supplementary Figure S6: Alpha-diversity indexes. Supplementary Figure S7: Quantification of *B. pseudolongum* and *R. ilealis *in the gut microbiota of FMT-CRC *Apc*^Min/+^mice. Supplementary Figure S8: Quantification of SCFA in the colon of FMT-CRC *Apc*^Min/+^mice supplemented with*A. inops *and *B. pseudolongum*. Supplementary Figure S9: Colonic cytokines levels in FMT-CRC mice. Supplementary Table S1: Demographic, clinical, and perioperative data of patients with colorectal cancer and healthy controls Supplementary Table S2: Erythroid parameters of patients with colorectal cancer. Supplementary Table S3: Primers for real-time PCR. Supplementary Table S4: Significant changes in relative abundances of microbial taxa at the family level.

## Data Availability

All raw sequence data generated and analyzed during the current study are available in the NCBI SRA repository [BioProject PRJNA1138753].
