# Supplementary material for: Initial gut microbiota composition is a determining factor in the promotion of colorectal cancer by oral iron supplementation: evidence from a murine model
Source: Microbiome. 2025 Apr 21;13:100. doi: 10.1186/s40168-025-02101-1 (PMC12013013; doi:10.1186/s40168-025-02101-1)
Supplement: Supplementary file 2 — Additional file 1. Supplementary Figure S1. Dietary iron supplementation promotes duodenal tumorigenesis in ApcMin/+mice independently of fecal microbiota transplant origin. Supplementary Figure S2: Representative pictures of the colon of ApcMin/+mice.Supplementary Figure S3: CRC associated gut microbiota differ from healthy control gut microbiota. Supplementary Figure S4: Bray-Curtis distances. Supplementary Figure S5: Beta diversity analysis of FMT-HC and FMT-CRC as a function of the dietary iron and initial gut microbiota composition. Supplementary Figure S6: Alpha-diversity indexes. Supplementary Figure S7: Quantification of B. pseudolongum and R. ilealis in the gut microbiota of FMT-CRC ApcMin/+mice. Supplementary Figure S8: Quantification of SCFA in the colon of FMT-CRC ApcMin/+mice supplemented withA. inops and B. pseudolongum. Supplementary Figure S9: Colonic cytokines levels in FMT-CRC mice. Supplementary Table S1: Demographic, clinical, and perioperative data of patients with colorectal cancer and healthy controls Supplementary Table S2: Erythroid parameters of patients with colorectal cancer. Supplementary Table S3: Primers for real-time PCR. Supplementary Table S4: Significant changes in relative abundances of microbial taxa at the family level. [file 40168_2025_2101_MOESM1_ESM.docx]

**Supplementary Material**

**Supplementary Figures**


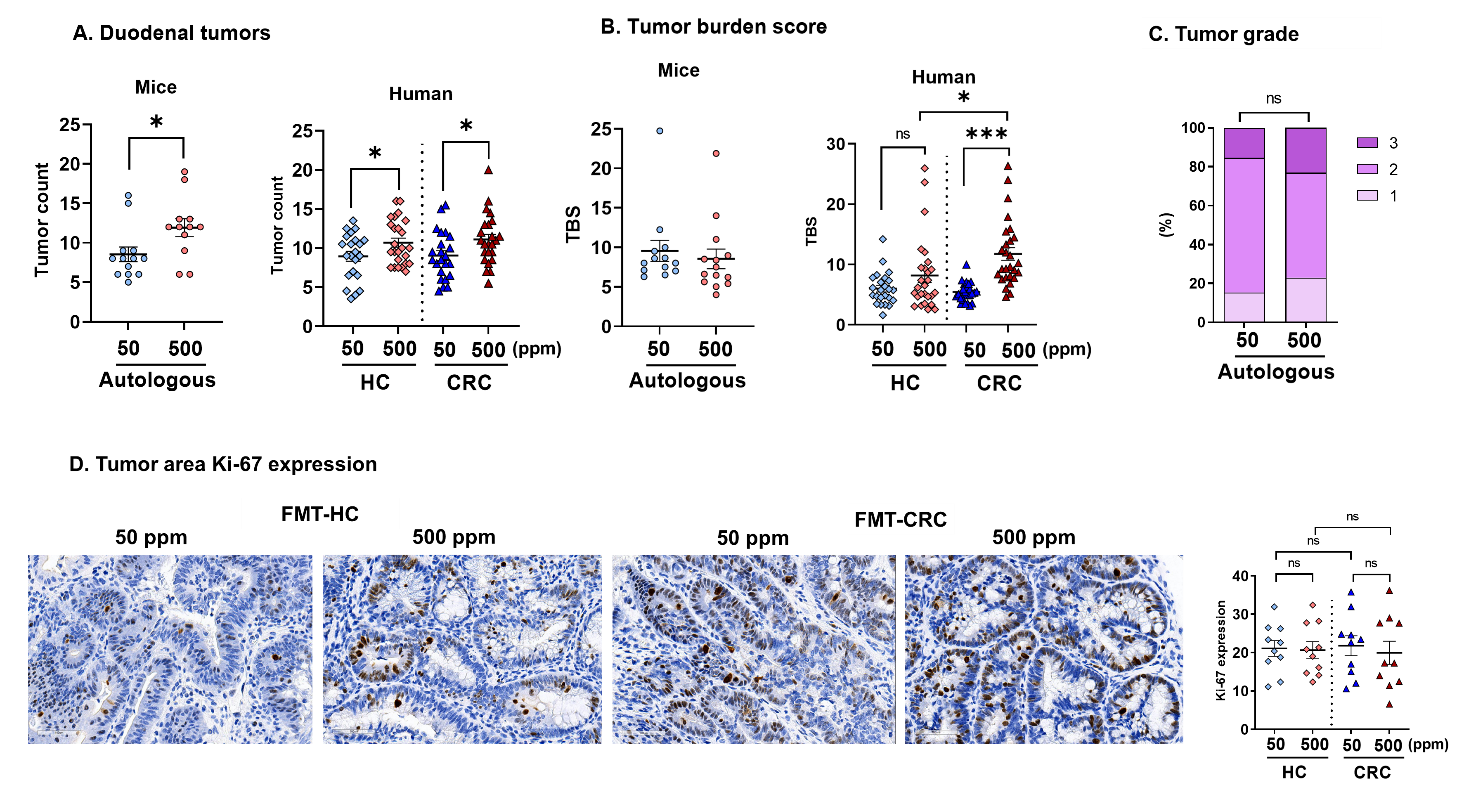


**Supplementary figure 1: Dietary iron supplementation promotes duodenal tumorigenesis in *Apc^M^*^in/+^ mice independently of fecal microbiota transplant origin.** (A) Duodenal tumor counts, (B) tumor burden, (C) tumor grade in auto-FMT mice and (D) immunohistochemical staining for Ki-67 quantification inside the tumor area. Each symbol represents one mouse, FMT-mice (n = 13 (50 ppm); n = 13 (500 ppm)), FMT-HC (n = 24 (50 ppm); n = 26 (500 ppm)) and FMT-CRC (n = 25 (50 ppm); n = 27 (500 ppm)). Bars are means ± SEM. *P* values were obtained using student *t*-test for FMT-mice. For mice transplanted with human fecal samples (FMT-HC and FMT-CRC) the generalized estimating equations (GEE) to correct for covariance structure of mice from a same donor (n= 2-3 mice/donor) was used. **P*<0.05. n.s.: non-significant.


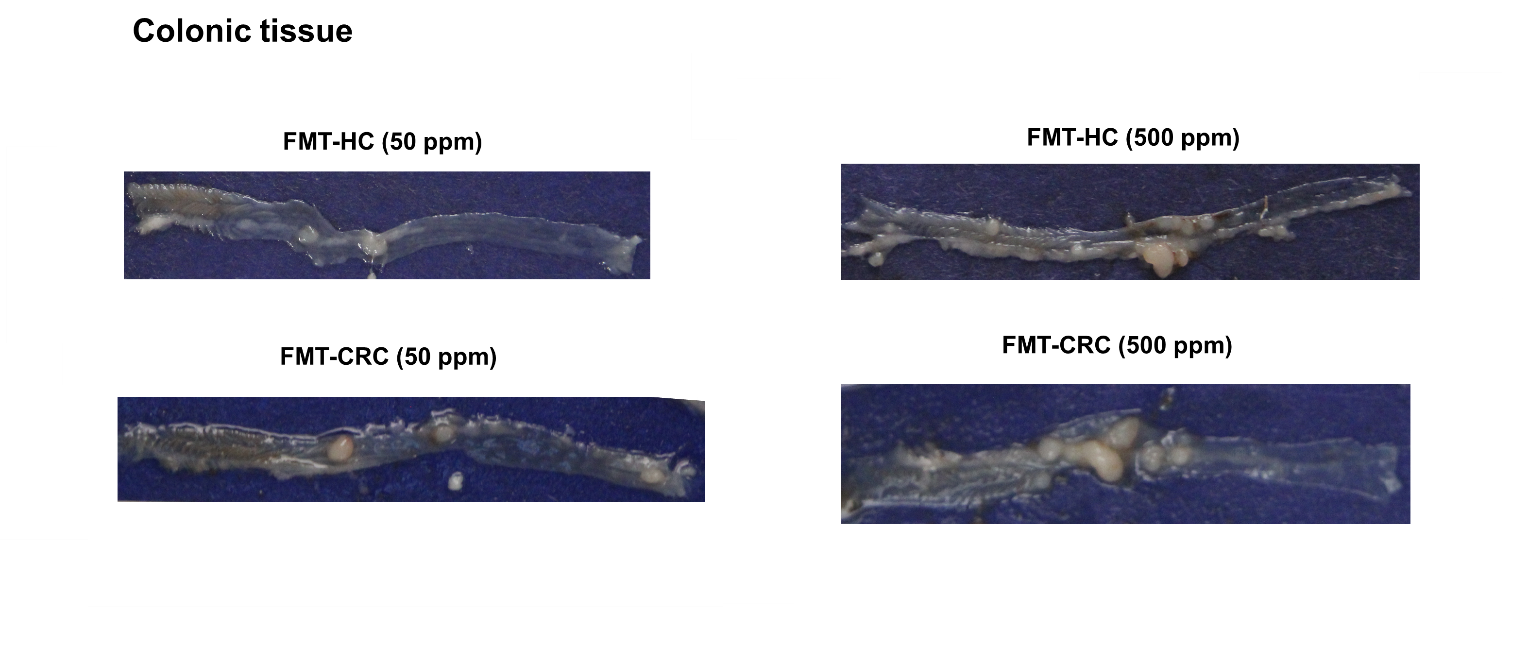
**Supplementary figure 2: Representative pictures of the colon of *Apc^M^*^in/+^ mice.**


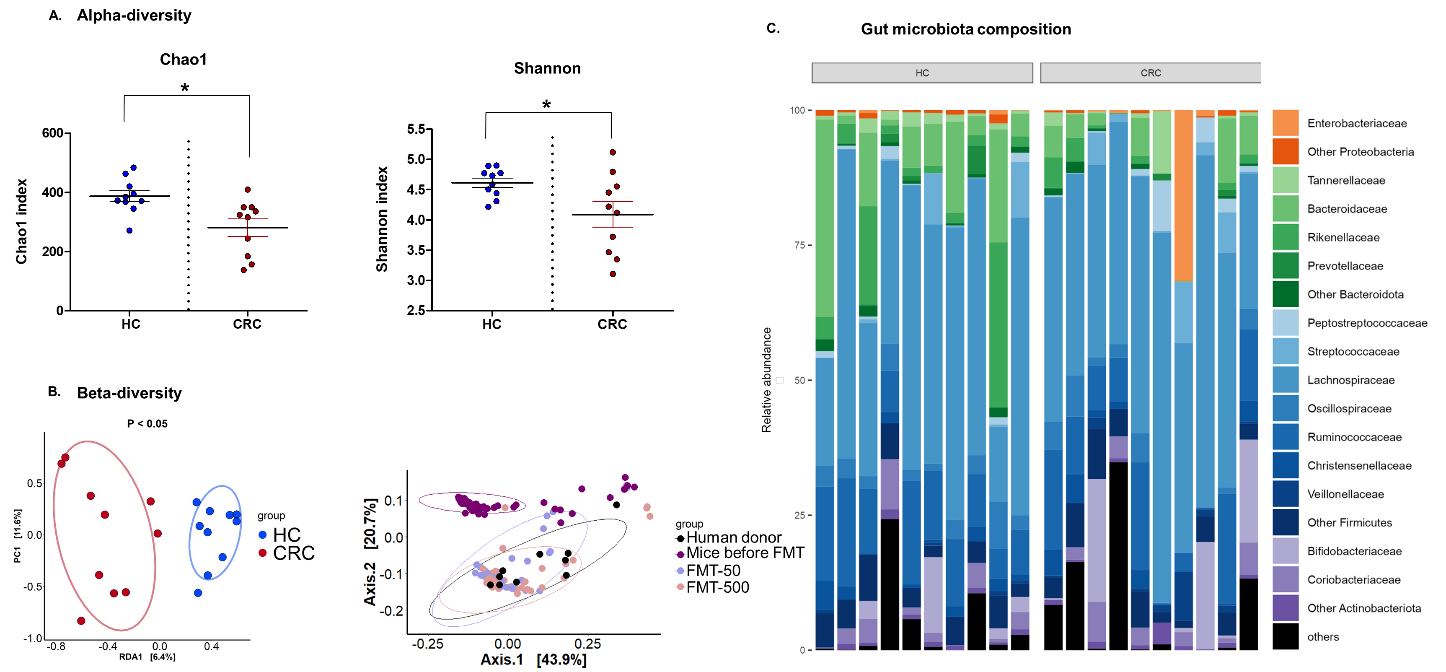


**Supplementary figure 3: CRC associated gut microbiota differ from healthy control gut microbiota.** (A) Alpha-diversity of the gut microbiota of healthy control and CRC patients **(**B) Distance-based redundancy analysis (RDA) of gut microbiota composition in human fecal samples (left) and weighted UniFrac of donors and mice pre and post FMT gut microbiota composition (right). (C). Relative abundances of microbial taxa at family levels. Bars are means ± SEM. HC n=10, CRC n=10. **P*<0.05. Student *t*-test.


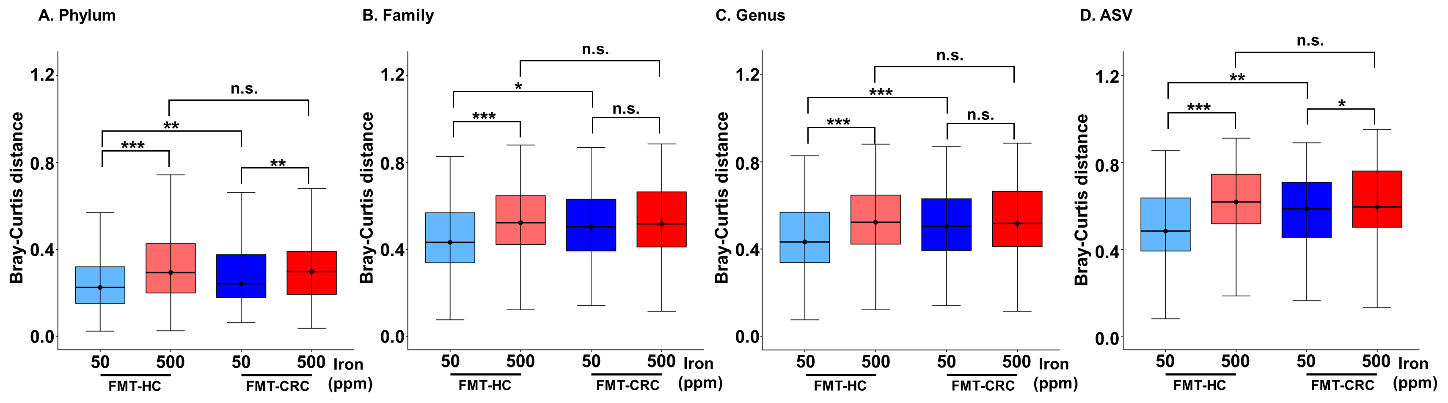


**Supplementary figure 4: Bray-Curtis distances.** Community dissimilarity at (A) Phylum, (B) Family, (C) Genus and (D) ASV levels within each experimental group. Box-and-whisker plots represent the median and interquartile range of Bray–Curtis distance between FMT-HC and FMT-CRC mice fed the iron sufficient or the iron excess diet. FMT-HC (n = 24 (50 ppm); n = 26 (500 ppm)) and FMT-CRC (n = 25 (50 ppm); n = 27 (500 ppm)). *P* values were obtained using Dunn’s Kruskal-Wallis Multiple Comparisons test. **P*<0.05, ***P*<0.01, ****P*<0.001, n.s.: non-significant.


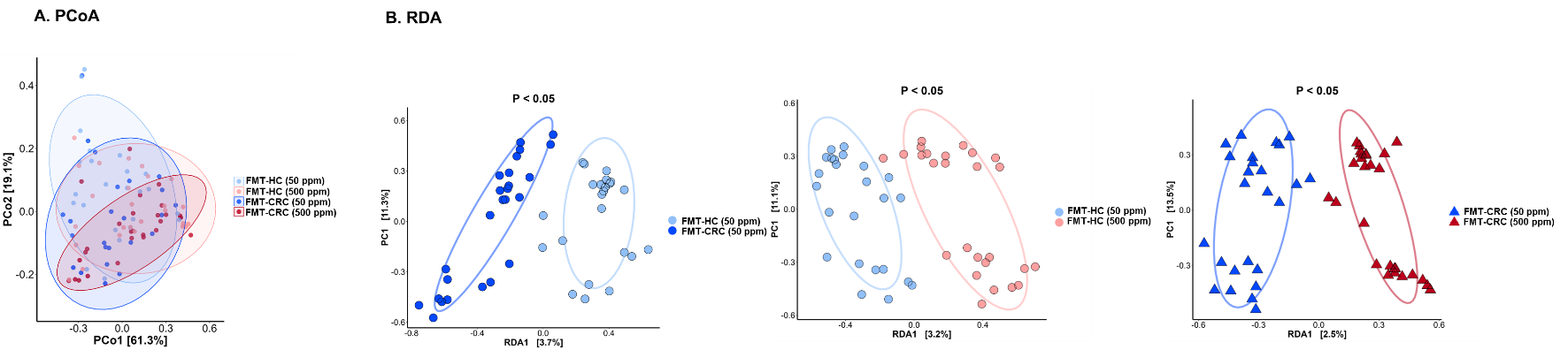


**Supplementary figure 5: Beta diversity analysis of FMT-HC and FMT-CRC as a function of the dietary iron and initial gut microbiota composition** (A) Weighted UniFrac distances and (B) Distance-based redundancy analysis (RDA) of gut microbiota composition in fecal samples comparing the effect of dietary iron between FMT-HC and FMT-CRC mice. Each symbol represents one mouse, FMT-mice (n = 13 (50 ppm); n = 13 (500 ppm)), FMT-HC (n = 24 (50 ppm); n = 26 (500 ppm)) and FMT-CRC (n = 25 (50 ppm); n = 27 (500 ppm)). **P*<0.05


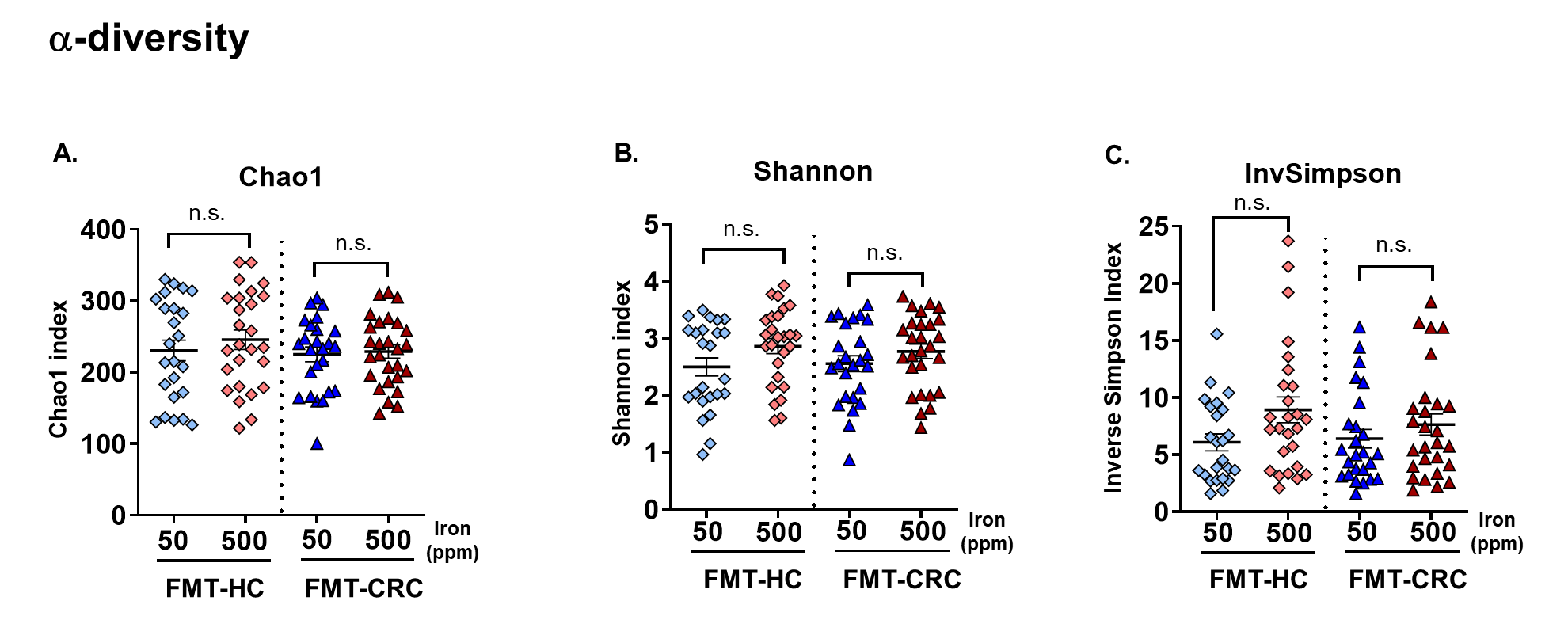


**Supplementary figure 6: alpha-diversity indexes.** (A) Chao1, (B) Shannon, and (C) inverse Simpson indexes of microbiota in fecal samples comparing the effect of dietary iron between FMT-HC and FMT-CRC mice. Each symbol represents one mouse. FMT-HC (n = 24 (50 ppm); n = 26 (500 ppm)) and FMT-CRC (n = 25 (50 ppm); n = 27 (500 ppm)). Bars are means ± SEM. *P* values were obtained using the generalized estimating equations (GEE) to correct for covariance structure of mice from a same donor (n= 2-3 mice/donor). n.s.: non-significant.


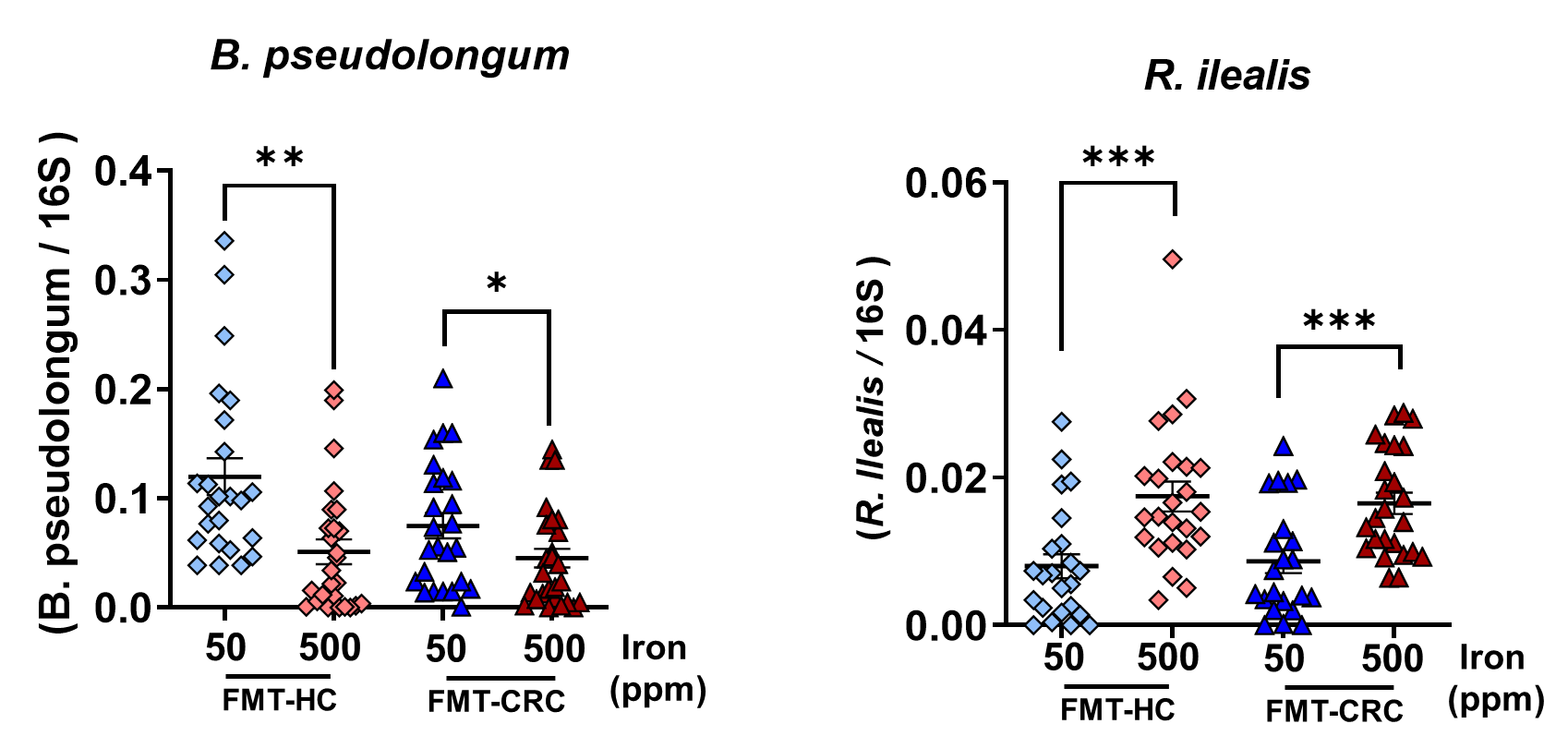


**Supplementary figure 7:** **Quantification of *B. pseudolongum* and *R. ilealis* in the gut microbiota of** **FMT-CRC** ***Apc^Min/+^* mice.** *Bifidobacterium pseudolongum* (left) and *Rombustia ilealis* (right) levels quantified by real-time PCR. Each symbol represents one mouse. FMT-HC (n = 24 (50 ppm); n = 26 (500 ppm)) and FMT-CRC (n = 25 (50 ppm); n = 27 (500 ppm)). *P* values were obtained using the generalized estimating equations (GEE) to correct for covariance structure of mice from a same donor (n= 2-3 mice/donor). Bars are means ± SEM. **P*<0.05, ***P*<0.01.


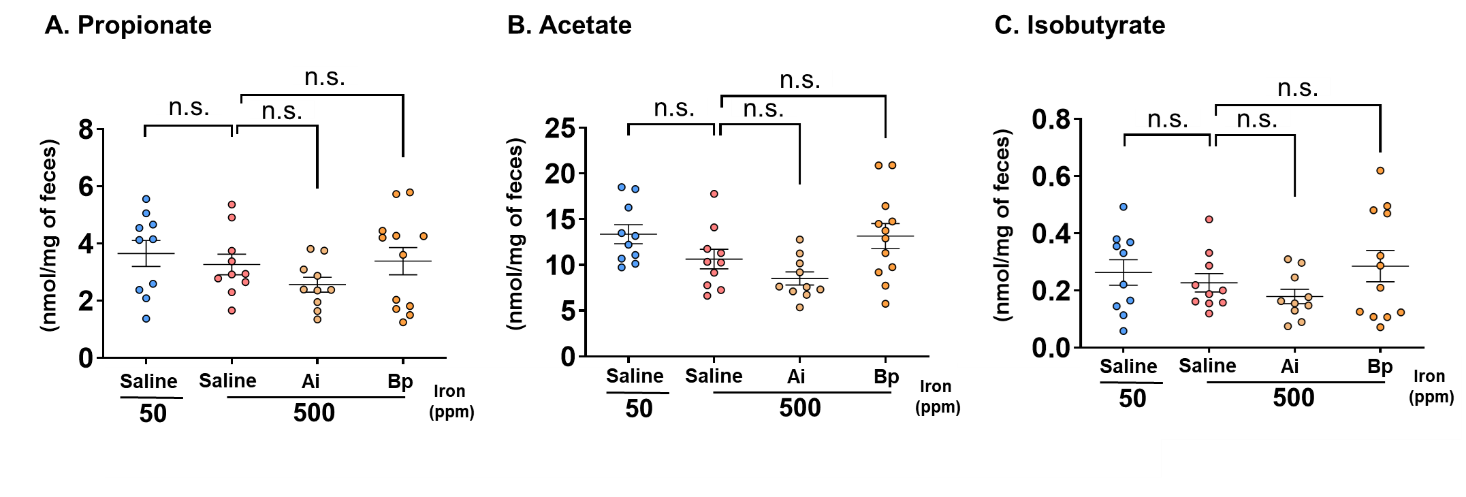


**Supplementary figure 8:** **Quantification of SCFA in the colon of** **FMT-CRC** ***Apc^Min/+^* mice supplemented with *A. inops* and *B. pseudolongum*.** Fecal (A) propionate (B) acetate and (C) isobutyrate. Each symbol represents one mouse. Bars are means ± SEM (n = 10-12). *P* values were obtained using one way ANOVA and post-hoc Dunnett test. n.s.: non-significant.


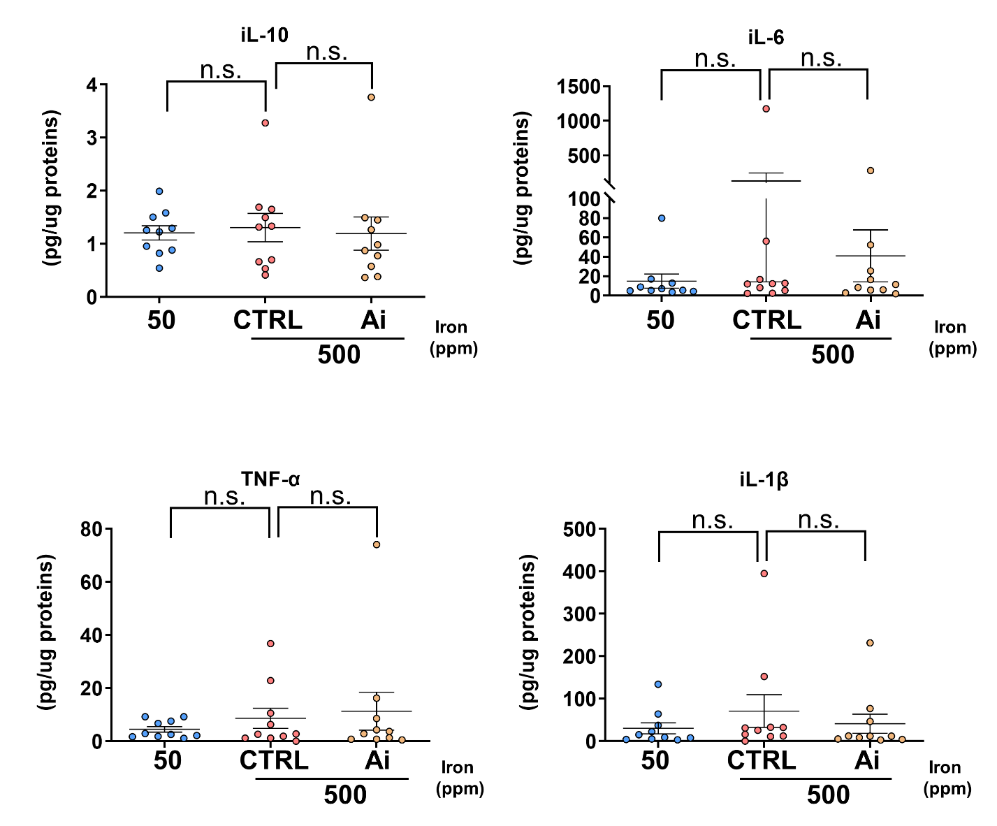


**Supplementary figure 9:** **Colonic cytokines levels in FMT-CRC mice**. The level of the following mucosal cytokines was assessed in FMT-CRC mice using a mouse cytokine multiplex assay: IL-10, IL-6, TNF-α and IL-1β. Each symbol represents one mouse. Bars are means ± SEM (n = 10-12). *P* values were obtained using one way ANOVA and post-hoc Dunnett test. n.s.: non-significant.

**Supplementary Table 1:** **Demographic, clinical, and perioperative data of patients with colorectal cancer and healthy controls**

| **Patients with colorectal cancer** | | | | | |
| --- | --- | --- | --- | --- | --- |
| **Patient ID** | **Sex** | **Age (years)** | **BMI (Kg/m^2^)** | **Cancer location** | **Grade** |
| 1 | F | 68 | 21.7 | Rectum | T3N+ |
| 2 | M | 58 | 25.2 | Recto-sigmoid | T2N0 |
| 3 | F | 76 | 33.2 | Caecum | T4N+M1 |
| 4 | M | 75 | 24.4 | Rectum | T3N0 |
| 5 | F | 68 | 26.7 | Descending Colon | T3N0 |
| 6 | M | 57 | 22.6 | Colon | T3N1b |
| 7 | M | 63 | 27.8 | Descending Colon | T3N+M+ |
| 8 | M | 49 | 25.8 | Sigmoid colon | T3N1a |
| 9 | F | 74 | 28.9 | Caecum | T3N0 |
| 10 | F | 58 | 34.4 | Descending Colon | T3N2aM1 |
| - | Mean ± SD | 64.0 ± 9.1 | 26.8 ± 4.2 | - | - |
| **Healthy controls** | | | | | |
| 11 | M | 66 | 24.2 | NA | NA |
| 12 | F | 62 | 19.3 | NA | NA |
| 13 | M | 59 | 25.6 | NA | NA |
| 14 | M | 68 | 32 | NA | NA |
| 15 | F | 66 | 26.5 | NA | NA |
| 16 | F | 71 | 34.2 | NA | NA |
| 17 | M | 66 | 27.5 | NA | NA |
| 18 | F | 66 | 30.9 | NA | NA |
| 19 | F | 71 | 28.7 | NA | NA |
| 20 | M | 65 | 29.1 | NA | NA |
| - | Mean ± SD | 65.9 ± 3.7 | 27.5 ± 4.2 | - | - |
| - | *P* values | n.s. | n.s. | - | - |

SD: standard deviation; BMI: body mass index; n.s.: non-significant

**Supplementary Table 2:** **Erythroid parameters of patients with colorectal cancer.**

| **Patient ID** | **RBC (x 10¹²/L)** | **Hemoglobin (g/L)** | **Hematocrit** | **MCV (fL)** |
| --- | --- | --- | --- | --- |
| 1 | 4.4 | 108 | 0.351 | 80.7 |
| 2 | 4 | 125 | 0.402 | 101.3 |
| 3 | 4.1 | 114 | 0.362 | 89.2 |
| 4 | 3.8 | 89 | 0.3 | 77.9 |
| 5 | 3.7 | 106 | 0.332 | 90.5 |
| 6 | 3.5 | 94 | 0.307 | 88 |
| 7 | 3.8 | 116 | 0.357 | 93.7 |
| 8 | 3.3 | 62 | 0.223 | 67.8 |
| 9 | 4.2 | 117 | 0.38 | 89.8 |
| 10 | 4.5 | 108 | 0.357 | 79.5 |

**Supplementary Table 3**: **Primers for real-time PCR**

| **Target** | **Sequence (5’-3’)** | | **Amplicon size (bp)** | **Reference** |
| --- | --- | --- | --- | --- |
| 16S rRNA gene | Forward | TCCTACGGGAGGCAGCAGTG | - | *Ettreiki et al.* (2012)^1^ |
|  | Reverse | TTACCGCGGCTGCTGGCACG |  |  |
| *Alistipes inops* | Forward | GCCTCAGCGTCCGATATAGA | 161 | This study |
|  | Reverse | AGGTGAAATTCCGAGGCTCA |  |  |
| *Faecalibaculum rodentium* | Forward | CCGGGAATACGCTCTGGAAA | 123 | *Zagato et al.* (2020)^2^ |
|  | Reverse | GCCAACCAACTAATGCACCG |  |  |
| *Holdemanella biformis* | Forward | GCTAAGGCCATGAACATGGA | 463 | *Zagato et al.* (2020)^2^ |
|  | Reverse | GCCGTCCTCTTCTGTTCTC |  |  |
| *Bifidobacterium pseudolongum* | Forward | CAAGGCCATCAACTGGTTCA | 120 | *Kim et al.*  *(2020)*^3^ |
|  | Reverse | ACGTCGTGCTGCTCGAATGT |  |  |

**Supplementary Table 4: Significant changes in relative abundances of microbial taxa at the family level.**

| **Phylum** | **Family** | **50 vs 500 HC** | **50 vs 500 CRC** | **HC vs CRC 50** | **HC vs CRC 500** |
| --- | --- | --- | --- | --- | --- |
| Actinobacteriota | *Eggerthellaceae* | n.s. | n.s. | n.s. | <0.05 |
| Bacteroidota | *Muribaculaceae* | n.s. | <0.001 | n.s. | n.s. |
| Desulfobacterota | *Desulfovibrionaceae* | <0.001 | n.s. | n.s. | n.s. |
| Firmicutes | *Eubacteriaceae* | <0.001 | <0.001 | n.s. | n.s. |
| Firmicutes | *Clostridiaceae* | n.s. | <0.001 | n.s. | n.s. |
| Firmicutes | *Lactobacillaceae* | n.s. | n.s. | <0.05 | n.s. |
| Firmicutes | *Peptococcaceae* | <0.001 | <0.05 | n.s. | n.s. |
| Firmicutes | *Peptostreptococcaceae* | <0.01 | n.s. | n.s. | n.s. |
| Firmicutes | *Streptococcaceae* | <0.001 | n.s. | n.s. | n.s. |
| Patescibacteria | *Saccharimonadaceae* | <0.001 | n.s. | n.s. | n.s. |
| Proteobacteria | *Enterobacteriaceae* | <0.001 | <0.01 | n.s. | n.s. |
| Verrucomicrobiota | *Akkermansiaceae* | <0.001 | <0.001 | n.s. | n.s. |

HC: Heathy control, CRC: Colorectal cancer, 50: 50 ppm iron, 500: 500 ppm iron, n.s.: non-significant

**Supplementary References**

1. Ettreiki C, Gadonna-Widehem P, Mangin I, et al. Juvenile ferric iron prevents microbiota dysbiosis and colitis in adult rodents. World J Gastroenterol 2012;18:2619-29.

2. Zagato E, Pozzi C, Bertocchi A, et al. Endogenous murine microbiota member Faecalibaculum rodentium and its human homologue protect from intestinal tumour growth. Nat Microbiol 2020;5:511-524.

3. Kim HB, Kim E, Yang SM, et al. Development of Real-Time PCR Assay to Specifically Detect 22 Bifidobacterium Species and Subspecies Using Comparative Genomics. Front Microbiol 2020;11:2087.
